# Photocopying the genetic lottery: a 2-gene shortcut to clonal seeds in rapeseed

**DOI:** 10.1093/plcell/koag174

**Published:** 2026-06-11

**Authors:** Yu-Hung Hung

**Affiliations:** Assistant Features Editor, The Plant Cell, American Society of Plant Biologists, United States; Spearhead Bio, Inc., 975 N. Warson Rd., St.Louis, MO 63132, United States

In the world of commercial agriculture, breeding elite F1 hybrid plants is like winning a lottery ticket. Through a phenomenon called *hybrid vigor*, or heterosis, these plants (carefully crossed from inbred lines) exhibit superior growth, stress resistance, and yield. However, nature's “casino dealer,” meiosis, ensures that this winning genetic combination is inevitably shuffled during seed production. For farmers, this means that saving seeds from these elite plants for the next season is a losing bet; the second-generation (F2) offspring will lack the uniform excellence of their mother plant. To “photocopy” that winning ticket, scientists have long pursued synthetic apomixis: a way to produce seeds that are perfect genetic clones of the mother plant (Mieulet et al. 2016; Xiong et al. 2023).

A primary tool for synthetic apomixis is the MiMe (Mitosis instead of Meiosis) system. In simple model plants like Arabidopsis, MiMe requires the simultaneous knockout of 3 specific genes: *SPO11-1* (the molecular “scissor” for DNA swapping), *REC8* (the glue for sister chromatids), and *OSD1* (the “emergency brake” that skips the second meiotic division) (d’Erfurth et al. 2009). While effective in diploids, the requirement for a 3-gene knockout makes it extremely challenging to replicate the MiMe system in complex polyploid crops such as rapeseed (*Brassica napus*). As an allotetraploid, *B. napus* contains redundant copies of genes across its A and C subgenomes, making the creation of triple mutants labor-intensive. Furthermore, mutating *OSD1* in rapeseed is deleterious, leading to severely stunted plants and developmental defects.

In a Breakthrough Report recently published in *The Plant Cell*, **Miaowei Geng and colleagues** demonstrate a remarkably simplified path forward (Geng et al. 2026). By targeting only 2 genes, *SPO11-1* and *REC8*, the team successfully converted meiosis into a mitosis-like division in *B. napus*. Surprisingly, they found that leaving the *OSD1* gene untouched did not ruin the clone. Cytological imagery revealed that in the absence of REC8 as glue, sister chromatids separated prematurely. This early separation apparently “short-circuits” the plant's internal biological clock, causing it to skip the second meiotic division naturally. Instead of the usual 4 shuffled cells (tetrads), the researchers observed dyads: 2 perfectly cloned cells (Fig. 1).

**Figure 1.**
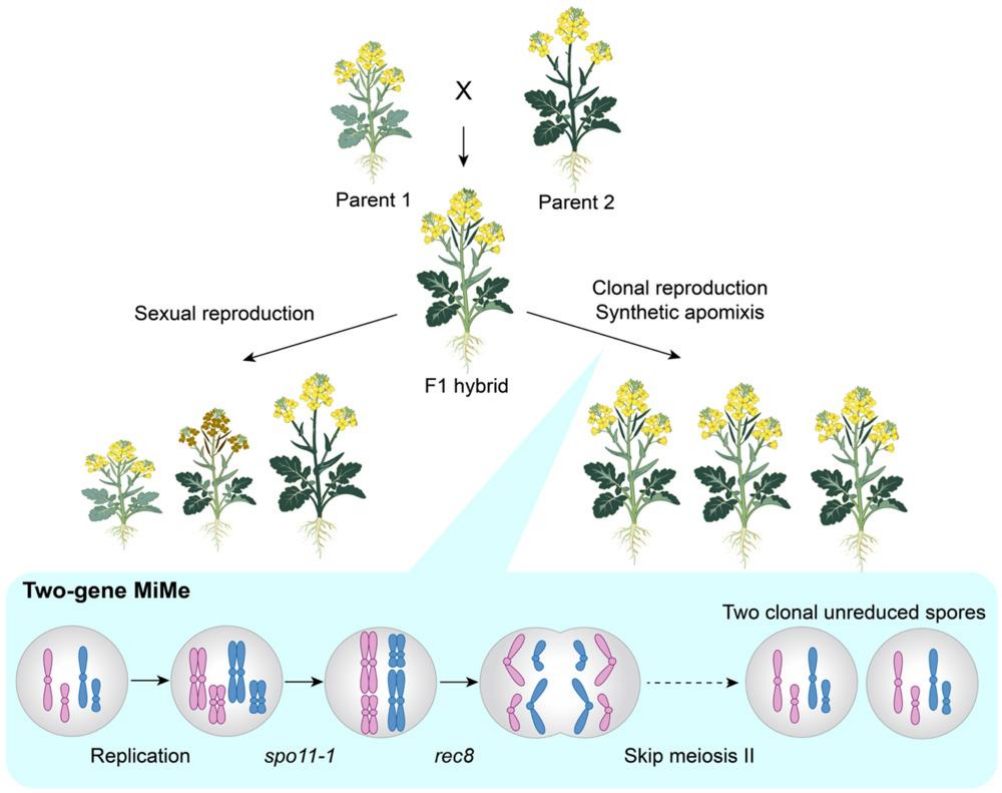
A 2-gene MiMe system for producing clonal gametes in rapeseed. A simplified 2-gene MiMe system that exploits the plant's polyploid nature to skip the second meiotic division in allotetraploid *Brassica napus*. Figure created with FigureLabs and Illustrator by Yu-Hung Hung.

To validate this “2-gene shortcut” strategy, the researchers used CRISPR/Cas9 to simultaneously knock out *REC8* and a second gene responsible for DNA swapping, such as *SPO11-1* or *MTOPVIB*, in F1 hybrids of 2 rapeseed cultivars (*Westar* x *J9707*). Whole-genome sequencing of the resulting offspring confirmed the system's high fidelity: 13 out of 14 plants inherited the full parental heterozygosity flawlessly, effectively freezing hybrid vigor across generations.

However, a significant bottleneck remains for commercial application: seed yield. While the plants produced viable, “giant” cloned pollen, the average seed set fell from 26 seeds per pod in wild-type plants to just 1.74. Cytological staining revealed that the pollen tubes grew normally in the style (the stalk connecting a flower's stigma to its ovary) but were unable to penetrate the ovules. This suggests a breakdown of the chemical communication between the unreduced gametes, which may function as an ancient evolutionary “firewall” to prevent catastrophic chromosome doubling in nature.

Despite this hurdle, the study elucidates the genetic architecture required for synthetic apomixis in complex polyploid crops. By combining this simplified MiMe system with parthenogenesis, a process that allows an egg to develop into an embryo without fertilization, this study opens a new avenue toward engineering clonal seed production in polyploid crops.

## Recent related articles in *The Plant Cell:*

- Bennett et al. 2025: This review synthesizes historical insights from Arabidopsis research to demonstrate its transformative impact on crop development across various domains, including root architecture, floral patterning, and synthetic apomixis.
- Hu et al. 2026: This study establishes a temporally resolved cytological framework for female meiosis in Arabidopsis by developing a live-cell imaging system.

## Data Availability

No new data were generated or analysed in support of this research.

## Recent related articles in *The Plant Cell*

- (Bennett et al. 2025) This review synthesizes historical insights from Arabidopsis research to demonstrate its transformative impact on crop development across various domains, including root architecture, floral patterning, and synthetic apomixis
- (Hu et al. 2026) This study establishes a temporally resolved cytological framework for female meiosis in Arabidopsis by developing a live-cell imaging system.
